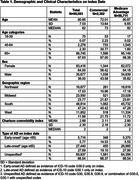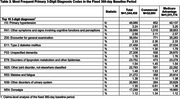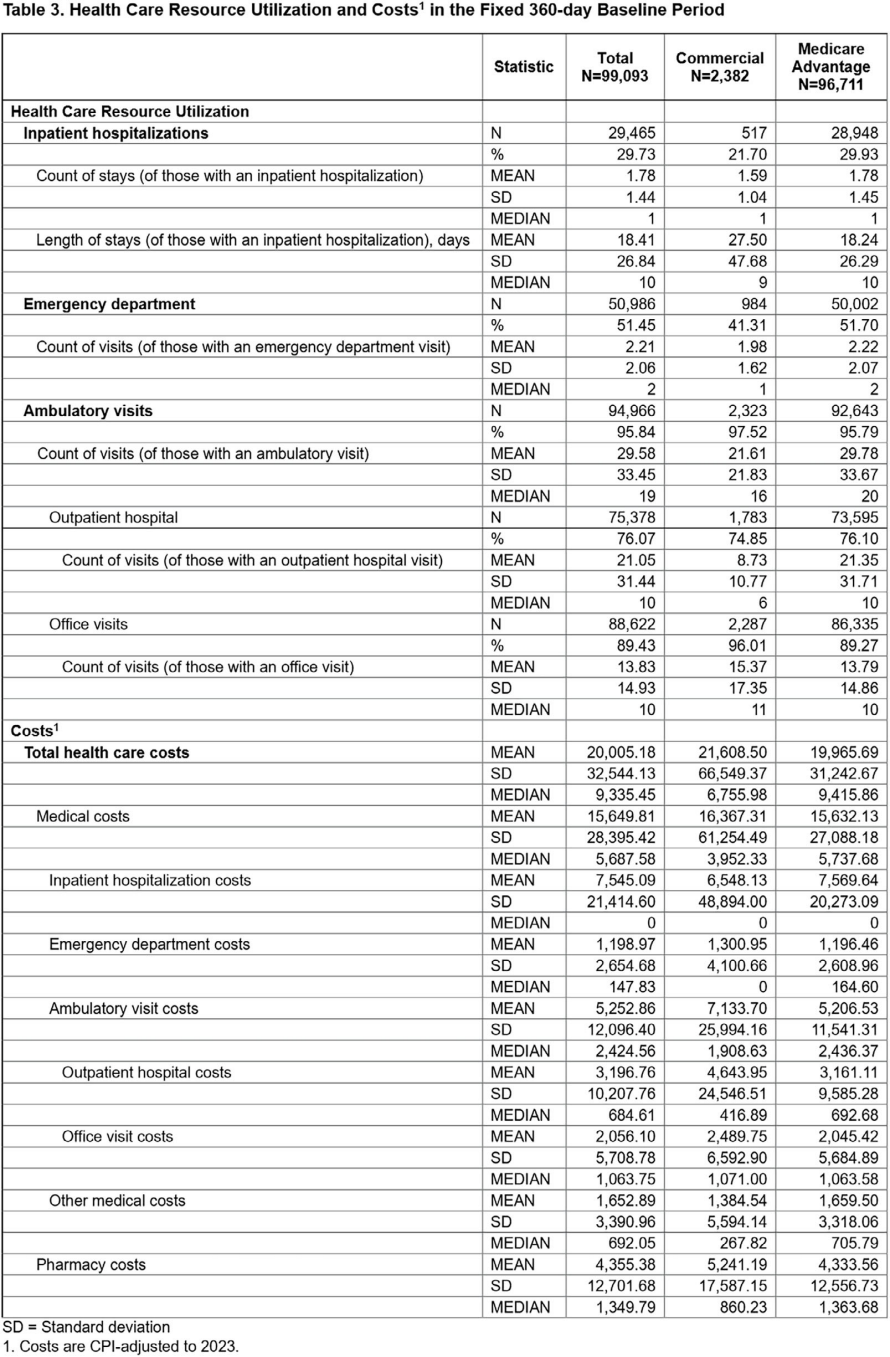# Clinical and health care characteristics preceding a new diagnosis of Alzheimer's disease in commercial and Medicare Advantage populations

**DOI:** 10.1002/alz70860_097112

**Published:** 2025-12-23

**Authors:** Julia M Certa, Jamie M Tucker, Pamela Morin

**Affiliations:** ^1^ Optum, Eden Prairie, MN, USA

## Abstract

**Background:**

As novel testing and treatments for Alzheimer's disease (AD) become more widely available, understanding the patient journey to diagnosis in real‐world settings is critical to adoption. This study aimed to identify a cohort of newly diagnosed AD patients and describe the clinical and health care characteristics leading up to diagnosis.

**Methods:**

De‐identified administrative claims data from the Optum Labs Data Warehouse were used to identify US adult commercial and Medicare Advantage enrollees 01/01/22‐03/31/24 with ≥1 claim for an AD diagnosis code (earliest claim = index date) and baseline enrollment ≥360 days. To identify newly diagnosed patients, those with evidence of AD in the 360‐day baseline period were excluded. Demographics, AD diagnosis type, and top provider specialties were captured on index. Comorbidities, top diagnoses, health care resource utilization and costs were captured in baseline.

**Result:**

Of 99,093 individuals newly diagnosed with AD, 97.6% were age ≥65, 64% were female, and 97.6% had Medicare Advantage insurance (Table 1). On index, provider specialty was most commonly primary care (35.0%) followed by hospital, neurology, psychology, and laboratory. AD diagnosis type was unspecified for the majority (66.5%) of patients, while 27.7% were diagnosed with late‐onset AD and 5.8% with early‐onset AD.

In the 360‐day baseline, top diagnosis codes included known AD risk factors (primary hypertension, type 2 diabetes) and more general conditions (Table 2). Nearly one‐third (29.7%) of patients had ≥1 inpatient hospitalization, with a mean stay of 18.4 days (standard deviation [SD] 26.8 days; Table 3). The majority (89.4%) of patients had ≥1 office visit, with an average of 13.8 visits (SD 14.9). Total health care costs were on average $20,005.18 (SD $32,544.13), primarily driven by inpatient hospitalization costs (mean $7,545.09, SD $21,414.60).

**Conclusion:**

In this claims‐based study, new AD diagnoses were most frequently associated with primary care providers and the AD type (early‐ vs. late‐onset) was unspecified. In the year before diagnosis, patients were seen for known AD risk factors as well as more general conditions, with extended inpatient hospital stays and frequent office visits. Future research is needed to assess evidence of biomarker testing and treatment post‐diagnosis.